# Early Screening for Confusion and Vitamin D Deficiency in Elderly Hip Fracture Patients: A Quality Improvement Initiative to Mitigate the Risk of Postoperative Delirium

**DOI:** 10.7759/cureus.75099

**Published:** 2024-12-04

**Authors:** Zubair Younis, Gurukiran Gurukiran, Faliq Abdullah, Sairam Kumar, David Ford, Muhammad A Hamid, Kubra Farooq Wani

**Affiliations:** 1 Orthopaedics, The Royal Wolverhampton NHS Trust, Wolverhampton, GBR; 2 Orthopaedics and Trauma, Royal Shrewsbury Hospital, Shrewsbury, GBR; 3 Orthogeriatrics, Royal Shrewsbury Hospital, Shrewsbury, GBR; 4 Orthopaedics and Trauma, The Robert Jones and Agnes Hunt Orthopaedic Hospital NHS Foundation Trust, Oswestry, GBR; 5 Orthopaedic Surgery, University Hospitals Birmingham NHS Foundation Trust, Birmingham, GBR; 6 Psychiatry, The Redwoods Centre, Shrewsbury, GBR

**Keywords:** cognitive screening, elderly patient care, hip fracture management, postoperative delirium, vitamin d deficiency

## Abstract

Background

Postoperative delirium (POD) is a common and debilitating complication in elderly hip fracture patients, associated with significant clinical and functional consequences. Early identification of risk factors, such as cognitive impairment and vitamin D deficiency, is essential to mitigate its impact. However, preoperative screening practices are often inconsistent. This quality improvement initiative aimed to assess and improve compliance with early confusion and vitamin D screening in elderly hip fracture patients, with the goal of facilitating timely interventions to reduce the risk of POD.

Methods

A two-cycle audit was conducted in the Trauma and Orthopaedics Department at Royal Shrewsbury Hospital. The first cycle (April-June 2023) assessed baseline compliance with confusion (Abbreviated Mental Test Score (AMTS)) and vitamin D screening within 24 hours of admission. Identified barriers informed an intervention consisting of educational sessions, visual prompts, and checklist integration. The second cycle (August-October 2023) evaluated the impact of these measures. Compliance rates, timing of assessments, and prevalence of abnormal biochemical and cognitive screening results were analyzed using Fisher’s exact test (p < 0.05).

Results

Baseline compliance with screening was 27 patients (27%) out of 100, with primary barriers including lack of awareness and logistical challenges. Post-intervention compliance improved significantly for 54 patients (52.4%) out of 103 (p = 0.0003). Screening timeliness also increased, with 45 patients (83.33%) out of 54 screenings completed within 24 hours in cycle 2 compared to 14 patients (51.85%) out of 27 in cycle 1 (p = 0.0039). Biochemical analysis revealed persistently high rates of vitamin D deficiency, underscoring the need for early detection. AMTS scores correlated with abnormal confusion screens but highlighted limitations in cognitive-only assessments.

Conclusion

This quality improvement (QI) initiative demonstrated the efficacy of targeted interventions in improving screening compliance for confusion and vitamin D deficiency in elderly hip fracture patients. The findings advocate for integrating proactive, dual-focused screening protocols into clinical workflows to address modifiable risk factors for POD. Future research should explore the long-term impact of such measures on POD incidence, recovery trajectories, and functional outcomes while emphasizing the need for sustained adherence to screening protocols.

## Introduction

In 1990, approximately 1.3 million hip fractures were reported worldwide, and this number is projected to increase to between 7.3 and 21.3 million by 2050 [1]. In England alone, over 89,000 older patients experience hip fractures each year [2]. Among all fractures in people aged 60 and above, hip fractures have the highest incidence and related costs [3]. For elderly patients, a hip fracture often marks a life-altering event, further diminishing their already potentially limited independence [4].

Comorbidities are common in patients with hip fractures, with 50% of cases occurring in individuals who already require nursing care [5]. Up to 20% of patients suffering from an osteoporotic hip fracture will die within the first year following the injury [6]. Postoperative delirium (POD), occurring in up to 54% of geriatric patients with hip fractures, is a significant complication in this population and is a proven independent risk factor for mortality, institutionalization, and dementia [7].

Preoperative risk factors for postoperative delirium (POD) include demographic factors like age and body mass index, pre-existing cognitive issues or mental disorders, physical health conditions such as other illnesses and American Society of Anesthesiologists (ASA) rating, and surgery-related factors like waiting time, hyponatremia, and blood pressure changes during surgery [8-11]. There are limitations to the identified risk factors for postoperative delirium (POD). First, factors like age and cognitive impairment may interact in ways that produce conflicting results, as aging doesn't always lead to significant cognitive decline. Second, risk factors should be linked to the underlying causes of POD, not just measured objectively. Lastly, assessments, such as cognitive tests, can be time-consuming and uncomfortable for patients, making them less frequently conducted before surgery [3].

In elderly patients with hip fractures, vitamin D deficiency is an important yet often overlooked factor impacting health outcomes. Low serum levels of 25-hydroxyvitamin D2 (vitamin D), with insufficiency defined as below 30 ng/mL and deficiency below 20 ng/mL, are associated with osteopenia, osteoporosis, and an increased risk of fractures [12]. Despite the well-established benefits of vitamin D and calcium supplementation, hypovitaminosis D remains an often overlooked and untreated condition, especially in the elderly population. Vitamin D deficiency contributes not only to poor musculoskeletal health but also to cognitive impairments, which can increase the risk of POD in patients with hip fractures [13].

While identifying high-risk factors for POD is crucial for early intervention, a more comprehensive approach may be necessary. Testing all patients over 60 for confusion and vitamin D deficiency could offer a more effective strategy for identifying those at greater risk and addressing underlying deficiencies preoperatively, potentially preventing POD in many cases. Early identification and intervention can help mitigate complications, improve recovery, and enhance overall patient outcomes.

This study aims to explore whether routine screening for confusion and vitamin D deficiency in hip fracture patients over 60 can identify those at high risk for POD, allowing for preoperative treatment to reduce the incidence of this debilitating complication.

## Materials and methods

This quality improvement project was conducted as a two-cycle audit in the Trauma and Orthopaedics Department at the Royal Shrewsbury Hospital, Shrewsbury, a district general hospital. The project aimed to assess compliance with early screening protocols for confusion and vitamin D deficiency in elderly trauma patients with hip fractures, implement an educational intervention to improve adherence, and evaluate the intervention’s effectiveness through a follow-up audit. The study included elderly patients (aged 60 and above) admitted with hip fractures requiring acute management. Patients with incomplete records or those transferred from other hospitals after the first 24 hours were excluded.

The audit, however, did not assess the direct impact of these screenings on clinical outcomes such as postoperative delirium (POD) incidence or long-term recovery.

Audit cycle 1: baseline assessment

In the first audit cycle, the medical records of 100 elderly trauma patients with hip fractures admitted between April and June 2023 were retrospectively reviewed. Data collection focused on whether confusion screening and vitamin D testing were completed within 24 hours of admission. Data collection included completion rates for the specified tests, timing relative to admission, and documented reasons for non-compliance when available. The audit also examined confusion screening using the Abbreviated Mental Test Score (AMTS) alongside biochemical parameters such as vitamin B12, folic acid, thyroid-stimulating hormone (TSH), and vitamin D. This analysis provided insights into the prevalence of abnormal values and the relationship between AMT scores and cognitive status.

Intervention 

After analyzing the results of the first audit cycle, an intervention was designed and implemented in July 2023 to address identified barriers to compliance. This included structured educational sessions for clinicians, nurses, and ancillary staff, focusing on the significance of early screening for confusion and vitamin D deficiency in elderly trauma patients. These sessions highlighted the connection between vitamin D levels, cognitive function, and the risk of postoperative delirium.

In addition to the educational sessions, visual materials such as posters outlining the screening protocols were strategically placed in prominent locations within the department. To reinforce the message, regular email reminders were circulated to healthcare providers, emphasizing the importance of timely assessments. To further streamline the process, the admission checklist for patients was updated to include prompts for confusion screening and vitamin D testing. This aimed to ensure that these assessments were prioritized within the first 24 hours of admission.

Audit cycle 2: post-intervention assessment 

A second audit cycle was conducted from August to October 2023 to measure the impact of the intervention. A total of 103 elderly trauma patients admitted during this period were assessed. Data collection and analysis mirrored that of cycle 1, focusing on the rates of confusion and vitamin D screening within 24 hours of admission.

Outcome measures 

The primary outcome was the rate of compliance with the early confusion and vitamin D screening protocols within the specified 24-hour window. Secondary outcomes included an analysis of the timing of assessments and any additional barriers to compliance.

Data collection and analysis

Data were collected from electronic patient records and documented in a standardized spreadsheet on Microsoft Excel (Microsoft Corp., Redmond, WA, USA) for each audit cycle. Compliance rates before and after the intervention were compared using Fisher’s exact test, with a significance level of p < 0.05. Additionally, qualitative feedback from staff about the intervention’s perceived impact was collected and summarized.

Ethical considerations

The study was conducted as part of a quality improvement initiative and did not require formal ethical approval. Patient confidentiality was maintained by anonymizing all data, and access to patient records was limited to the audit team.

## Results

Baseline data (audit cycle 1) 

During the first audit cycle, of the 100 patients reviewed, only 27 (27%) underwent confusion screening and vitamin D testing within the recommended 24-hour window. The primary barriers identified were lack of staff awareness regarding the importance of early testing, limited access to screening tools in high-demand areas, and the absence of routine reminders in clinical workflows.

Post-intervention data (audit cycle 2) 

Following the implementation of the educational intervention, compliance improved significantly. Of the 103 patients in the second audit cycle, 54 (52.4%) underwent confusion screening and vitamin D testing within 24 hours of admission. This represents an increase in compliance with a change that was statistically significant (Fisher’s exact test, p = 0.0003, significant at p < 0.05). This improvement highlights the effectiveness of education and visual reminders in increasing adherence to screening protocols (Table 1).

**Table 1:** Compliance with confusion and vitamin D screening in audit cycles The Fisher exact test statistic value is 0.0003. The result is significant at p < .05.

Results	Audit 1 N(%)	Audit 2 N(%)	Marginal Row Totals
Screened	27 (27%)	54 (52.4%)	81
Not screened	73 (73%)	49 (47.57%)	122
Marginal column totals	100	103	203 (Grand Total)

Timing of screening 

Further analysis of the timing of screenings in each audit cycle showed improvements in promptness post-intervention. In audit cycle 1, 14 (51.85%) out of the 27 patients who received screening did so within 24 hours of admission. By audit cycle 2, this figure had risen to 45 (83.33%) out of 54 patients, indicating both an increase in the number of screenings performed and an improvement in the timeliness of screening (Fisher’s exact test, p = 0.0039, significant at p < 0.05). These findings suggest that the intervention helped prioritize early testing in clinical workflows (Table 2).

**Table 2:** Timing of screening in audit cycles The Fisher exact test statistic value is 0.0039. The result is significant at p < .05.

Timing of Screening	Audit 1 N(%)	Audit 2 N(%)	Marginal Row Totals
Within 24 hours	14 (51.85%)	45 (83.33%)	59
After 24 hours	13 (48.14%)	9 (16.66%)	22
Marginal column totals	27	54	81 (Grand Total)

Biochemical and cognitive screening results

Biochemical screening revealed trends in abnormal vitamin B12, folic acid, thyroid stimulating hormone (TSH), and vitamin D levels. The proportion of patients with abnormal vitamin D levels remained high across both cycles, underscoring the importance of early detection and intervention (Table 3).

**Table 3:** Biochemical screening results in audit cycles TSH: thyroid stimulating hormone

Parameter	Audit Cycle	Normal Levels N (%)	Abnormal Levels N (%)
Vitamin B12	Audit 1	74 (74%)	26 (26%)
	Audit 2	72 (72%)	31 (31%)
Folic acid	Audit 1	62 (62%)	38 (38%)
	Audit 2	75 (75%)	29 (29%)
TSH	Audit 1	80 (80%)	20 (20%)
	Audit 2	84 (84%)	19 (19%)
Vitamin D	Audit 1	44 (44%)	56 (56%)
	Audit 2	48 (48%)	55 (55%)

Confusion screen outcomes relative to Abbreviated Mental Test Score (AMTS)

The analysis of AMT scores revealed consistent trends in cognitive screening outcomes.

Low Abbreviated Mental Test Scores (AMTS)

In audit cycle 1, 18 patients (42%) had normal confusion screens, and 25 patients (58%) showed abnormal results. These proportions remained unchanged in audit cycle 2.

Normal Abbreviated Mental Test Scores (AMTS)

In audit cycle 1, 20 patients (35%) had normal confusion screens, and 37 patients (65%) showed abnormal results. In audit cycle 2, 20 patients (36%) had normal confusion screens, while 35 patients (64%) showed abnormal results (Table 4).

**Table 4:** Confusion screen outcomes by AMT scores in audit cycles AMT: Abbreviated Mental Test

AMT Score	Audit Cycle	Normal Confusion Screen N (%)	Abnormal Confusion Screen N (%)
Low AMT Score	Audit 1	18 (42%)	25 (58%)
	Audit 2	20 (42%)	28 (58%)
Normal AMT Score	Audit 1	20 (35%)	37 (65%)
	Audit 2	20 (36%)	35 (64%)

Comparative analysis 

The results demonstrate that the educational intervention led to a statistically significant improvement in compliance with confusion and vitamin D screening guidelines. The increase in compliance from 27 patients (27%) to 54 patients (52.4%), along with improvements in timely completion, indicates that awareness strategies and visual reminders were effective in addressing initial barriers. Additionally, trends in AMT scores and biochemical abnormalities highlight the importance of early screening for identifying at-risk patients and improving clinical outcomes through timely intervention.

## Discussion

This quality improvement audit demonstrates the significant role of structured interventions in improving the early detection of confusion and vitamin D deficiency in elderly patients with hip fractures. Early screening is crucial in this population, where postoperative delirium (POD) is prevalent and strongly associated with increased mortality, morbidity, and long-term functional decline. By identifying and addressing correctable deficiencies before surgery, our audit supports the adoption of proactive screening protocols as a standard of care in orthopedic trauma settings to mitigate POD risk and possibly improve patient outcomes. 

The impact of early screening on postoperative delirium (POD) prevention

The findings emphasize the importance of early intervention to prevent the cascade of complications that can lead to POD. Preoperative serum vitamin D levels have been shown to be associated with postoperative delirium after major surgery, highlighting the need for early detection and correction of deficiencies [14]. Cycle 1 revealed a compliance rate of only 27 patients (27%) in early screening for confusion and vitamin D deficiency, highlighting a significant gap in preoperative management. Despite established guidelines recommending these screenings in patients with low AMTS, barriers, including limited staff awareness, time constraints, and restricted access to screening tools, can pose obstacles to performing assessments within the 24-hour post-admission window. This trend aligns with common challenges in orthogeriatric care, where immediate cognitive and biochemical evaluations can sometimes be difficult.

Delays in completing preoperative screenings not only reduce compliance rates but also risk prolonging the time to surgery. The association between surgical delay and preoperative delirium underscores the urgency of timely intervention. Addressing logistical challenges to ensure that confusion and biochemical screening are completed within 24 hours post-admission can minimize surgical delays [15]. Prolonged waiting times, often caused by incomplete assessments or logistical issues, increase the risk of preoperative delirium and postoperative complications, prolonging hospital stays and impairing recovery trajectories. Studies have demonstrated greater mortality if the preoperative waiting time exceeds 48 hours, and surgical delay has also been associated with a greater risk of pressure ulcers and infectious complications and longer length of stay [16,17].

Our educational intervention aimed to bridge these gaps by enhancing staff training, reinforcing the importance of early screenings, and introducing visual reminders and checklists. These measures proved effective, as evidenced by a post-intervention compliance increase to 54 patients (52.4%). The improvement not only demonstrates the potential for enhancing patient care through systematic prompts and focused education but also underscores that proactive, timely screening can be effectively integrated into clinical workflows, even in high-demand environments. The statistically significant improvement (p = 0.0039) supports the hypothesis that early correction of deficiencies may reduce POD risk, as prompt vitamin D supplementation and cognitive interventions target key modifiable risk factors before surgery.

Delirium's independent impact on functional recovery

Delirium is independently associated with poor functional recovery after hip fractures, even after adjusting for pre-fracture frailty factors such as advanced age and baseline cognitive impairment [18]. This observation aligns with existing literature emphasizing that persistent delirium exacerbates declines in activities of daily living (ADL) and ambulation, leading to higher mortality and nursing home placements [19,20]. Furthermore, the concept of a "dose-response" effect, where persistent delirium leads to worse outcomes than transient cases, underscores the importance of prompt, targeted interventions [18].

Early screening and correction of modifiable risk factors, such as vitamin D deficiency and cognitive impairment, can help intercept the trajectory of delirium. By addressing these issues preoperatively, our initiative aligns with calls for preemptive measures that aim to mitigate the long-term functional decline associated with POD.

Abbreviated Mental Test Score as predictive markers and their limitations in forecasting postoperative delirium risk

An essential element of our audit was examining the role of AMTS scores in predicting POD risk. Our analysis revealed that low AMT scores were associated with higher rates of abnormal confusion screening outcomes, reinforcing that cognitive impairment is a known risk factor for POD. However, findings also indicated that even patients with normal AMT scores exhibited high rates of abnormal confusion screens, suggesting that AMT alone may have limited predictive value in determining which patients are at greater risk of adverse cognitive outcomes. 

The limited predictive ability of AMT scores highlights a need for a more comprehensive approach to risk assessment that goes beyond cognitive scoring alone. By pairing AMT scores with biochemical assessments, clinicians can adopt a dual-focused approach that addresses both cognitive and biochemical vulnerabilities. Identifying patients with low AMT scores and concurrent vitamin D deficiency may provide a more targeted means of guiding preoperative interventions and help reduce the overall incidence of POD. This approach aligns with emerging evidence linking vitamin D deficiency to neuromuscular dysfunction, poor bone health, and impaired cognitive recovery, underscoring the need to prioritize its correction as a key step in POD prevention [13]. 

Implications for clinical practice and future quality improvement

This initiative demonstrates that systematic, proactive screening protocols are both feasible and beneficial in elderly patients with hip fractures. Educational interventions, checklists, and visual prompts can drive substantial improvements in compliance, even in high-demand clinical settings. Future efforts should explore integrating automated reminders into electronic health records and adopting bedside cognitive screening tools to standardize assessments.

Longitudinal studies are also recommended to evaluate the long-term impact of early screening on POD incidence and recovery outcomes. Investigating optimal timing and combinations of interventions, including vitamin D supplementation and cognitive support, will provide robust evidence for refining screening protocols.

This quality improvement audit has several limitations. First, it was conducted in a single center, which may limit the applicability of the findings to other healthcare settings with different patient demographics or resources. The retrospective nature of the study also introduces the risk of selection bias, as patient records may have been incomplete or inconsistently documented. While the intervention improved compliance with screening protocols, we did not evaluate the long-term sustainability of these changes beyond the audit period. Additionally, although confusion and vitamin D screening improved, the audit did not assess the direct impact of these screenings on clinical outcomes such as postoperative delirium (POD) incidence or long-term recovery. The reliance on AMT scores as a cognitive assessment tool may also limit the comprehensiveness of cognitive function assessment, potentially underestimating cognitive impairment in some patients.

## Conclusions

This quality improvement initiative highlights the importance of early screening for confusion and vitamin D deficiency in elderly hip fracture patients. The intervention effectively improved compliance with screening protocols through educational sessions, visual prompts, and checklists. While AMT scores were useful for identifying cognitive impairment, a more comprehensive screening approach is recommended to better address postoperative delirium risk.

Future efforts should focus on sustaining improved compliance rates and exploring the long-term impact of early interventions on patient outcomes. Enhancing screening workflows with automated prompts and advanced cognitive tools may further optimize care for this vulnerable population.

## References

[1] Gullberg B, Johnell O, Kanis JA (1997). World-wide projections for hip fracture. Osteoporos Int.

[2] (2024). Guidance For Older Patients With Hip Fractures. https://www.boa.ac.uk/static/267d3fa8-3515-4ff5-9e14611eabeee018/guidance-for-older-patients-with-hip-fractures-18.3.13.pdf.

[3] Zhao S, Sun T, Zhang J, Wang Y, Guo Y, Wang X (2024). How to predict postoperative delirium in geriatric patients with hip fracture as soon as possible? A retrospective study. BMC Surg.

[4] Fischer H, Maleitzke T, Eder C, Ahmad S, Stöckle U, Braun KF (2021). Management of proximal femur fractures in the elderly: current concepts and treatment options. Eur J Med Res.

[5] Rapp K, Büchele G, Dreinhöfer K, Bücking B, Becker C, Benzinger P (2019). Epidemiology of hip fractures: systematic literature review of German data and an overview of the international literature. Z Gerontol Geriatr.

[6] Leibson CL, Tosteson AN, Gabriel SE, Ransom JE, Melton LJ (2002). Mortality, disability, and nursing home use for persons with and without hip fracture: a population-based study. J Am Geriatr Soc.

[7] Bruce AJ, Ritchie CW, Blizard R, Lai R, Raven P (2007). The incidence of delirium associated with orthopedic surgery: a meta-analytic review. Int Psychogeriatr.

[8] Wu J, Yin Y, Jin M, Li B (2021). The risk factors for postoperative delirium in adult patients after hip fracture surgery: a systematic review and meta-analysis. Int J Geriatr Psychiatry.

[9] Fortes-Filho SQ, Apolinario D, Melo JA, Suzuki I, Sitta Mdo C, Garcez Leme LE (2016). Predicting delirium after hip fracture with a 2-min cognitive screen: prospective cohort study. Age Ageing.

[10] Juliebø V, Bjøro K, Krogseth M, Skovlund E, Ranhoff AH, Wyller TB (2009). Risk factors for preoperative and postoperative delirium in elderly patients with hip fracture. J Am Geriatr Soc.

[11] Freter S, Dunbar M, Koller K, MacKnight C, Rockwood K (2016). Prevalence and characteristics of pre-operative delirium in hip fracture patients. Gerontology.

[12] Holick MF (2007). Vitamin D deficiency. N Engl J Med.

[13] Boonen S, Rizzoli R, Meunier PJ (2004). The need for clinical guidance in the use of calcium and vitamin D in the management of osteoporosis: a consensus report. Osteoporos Int.

[14] Tumer NB, Tekeli Kunt A, Gunaydin S, Ozisik K (2020). Preoperative vitamin D level is associated with postoperative delirium after cardiac surgery in patients over 65 years of age. Heart Surg Forum.

[15] Friedman SM, Mendelson DA, Kates SL, McCann RM (2008). Geriatric co-management of proximal femur fractures: total quality management and protocol-driven care result in better outcomes for a frail patient population. J Am Geriatr Soc.

[16] Grimes JP, Gregory PM, Noveck H, Butler MS, Carson JL (2002). The effects of time-to-surgery on mortality and morbidity in patients following hip fracture. Am J Med.

[17] Verbeek DO, Ponsen KJ, Goslings JC, Heetveld MJ (2008). Effect of surgical delay on outcome in hip fracture patients: a retrospective multivariate analysis of 192 patients. Int Orthop.

[18] Marcantonio ER, Flacker JM, Michaels M, Resnick NM (2000). Delirium is independently associated with poor functional recovery after hip fracture. J Am Geriatr Soc.

[19] Inouye SK, Rushing JT, Foreman MD, Palmer RM, Pompei P (1998). Does delirium contribute to poor hospital outcomes? A three-site epidemiologic study. J Gen Intern Med.

[20] Murray AM, Levkoff SE, Wetle TT (1993). Acute delirium and functional decline in the hospitalized elderly patient. J Gerontol.

